# Finite Element Analysis of Cornea and Lid Wiper during Blink, with and without Contact Lens

**DOI:** 10.1155/2022/7930334

**Published:** 2022-05-17

**Authors:** Vivek Suganthan Ramasubramanian, S. Meenatchi Sundaram, Rinu Thomas, S. Ve. Ramesh, B. Raghuvir Pai, Manali Hazarika, Shah Mohammed Abdul Khader, Rakshath G. Poojary, H. Girish, Vernon Seth Crasto

**Affiliations:** ^1^Department of Optometry, Manipal College of Health Professions, Manipal Academy of Higher Education, Manipal 576104, India; ^2^Department of Instrumentation and Control Engineering, Manipal Institute of Technology, Manipal Academy of Higher Education, Manipal 576104, India; ^3^Department of Mechanical and Manufacturing Engineering, Manipal Institute of Technology, Manipal Academy of Higher Education, Manipal 576104, India; ^4^Department of Ophthalmology, Kasturba Medical College, Manipal Academy of Higher Education, Manipal 576104, India

## Abstract

Ocular surface disorders such as Lid Wiper Epitheliopathy (LWE), Superior Epithelial Arcuate Lesion (SEAL), and contact lens-induced Limbal Stem Cell Deficiency (LSCD) as well as Superior Limbic Keratoconjunctivitis (SLK) affect one's quality of life. Hence, it is imperative to investigate the underlying causes of these ocular surface disorders. During blink, the undersurface of the eyelid tends to interact with the cornea and the conjunctiva. The presence of a contact lens can add to the biomechanical frictional changes on these surfaces. To estimate these changes with and without a contact lens, a finite element model (FEM) of the eyelid wiper, eyeball, and contact lens was developed using COMSOL Multiphysics. Biomechanical properties such as von Mises stress (VMS) and displacement were calculated. Our study concluded that (a) maximum VMS was observed in the lid wiper in the absence of contact lens in the eye and (b) maximum VMS was observed in the superior 1.3 mm of the cornea in the presence of the contact lens in the eye. Thus, the development of friction-induced ocular surface disorders such as LWE, SLK, SEAL, and LSCD could be attributed to increased VMS. FEA is a useful simulation tool that helps us to understand the effect of blink on a normal eye with and without CL.

## 1. Introduction

It is estimated that currently there are more than 140 million contact lens (CL) wearers worldwide [1]. Existing literature states that about 21%–64% of these CL wearers tend to discontinue CL use [2–4] as a result of contact lens discomfort (CLD). CLD is a condition characterized by “episodic or persistent adverse ocular sensations due to CL wear, either with or without visual disturbance. It arises from a reduced compatibility between the CL and the ocular environment and thus accounts for 50% of patients' experiencing a reduced CL-wearing time and/or discontinuation of CL use” [5]. Factors contributing to CLD include CL material, design, lens care, fit, and tear film stability [6]. Apart from CLD, dry eye (19.9%) [7], ocular redness (6.8%) [7], cost of CL (6.8%) [7], and handling issues (6.3%) [7] can also lead to CL dropouts. Conditions such as age; gender; systemic diseases like autoimmune diseases; CL solution and care regimen; and design, material, and modality of the CL account for a smaller percentage of CL dropouts [1, 8].

During the blink reflex, the undersurface of the eyelid interacts with the cornea and the conjunctiva [9]. This setup of the human eye can be considered as a complex tribological system consisting of two sliding surfaces (eyelid and corneo-conjunctival complex) moving relative to each other, with the tear film as a lubricant [10]. Deficiency of tear fluid or excessive evaporation of tears may lead to dry eye disease [11] which in turn may harm the ocular surface due to friction [12]. Having a CL *in situ* results in two sets of sliding surfaces. While in the anterior aspect, the sliding surfaces are is formed by the palpebral conjunctiva (which lines the inner surface of the eyelid) and anterior surface of the contact lens, in the posterior aspect, it is formed by the posterior surface of contact lens, anterior surface of the cornea, and bulbar conjunctiva [13]. This tribological system is also highly influenced by various tear film characteristics that may result from the presence of dry eye disease in CL wearers.

The cornea and the lid wiper (a portion of the lid margin of the upper eyelid that wipes the ocular surface during blinking) [14] are more prone to frictional damage than the bulbar conjunctiva [15]. Frictional damage to the ocular surface causes ocular surface disorders such as Lid Wiper Epitheliopathy (LWE), Superior Limbic Keratoconjunctivitis (SLK) [16], Superior Epithelial Arcuate Lesion (SEAL) [17], and Limbal Stem Cell Deficiency (LSCD) [18]. These conditions cause a variety of symptoms such as foreign-body sensation during blink, discomfort, and visual loss, thereby decreasing the quality of life [16].

Friction is usually quantified using Coefficient of Friction (CoF) which is the ratio between the frictional force and the normal force. The most commonly used *in vitro* methods to measure CoF are through the use of an instrument called micro tribometer [19, 20] and atomic force microscopy [21, 22]. In case of CL, CoF can vary depending on its material properties, thickness, and modulus of elasticity. However, in general, a CL having low CoF reduces friction and improves comfort [10, 20, 23–25]. The CoF values range from 0.011 to 0.562 across various studies [19, 20, 26]. This wide variation noted is due to the difference in the material used to measure the CoF. For example, instead of cornea and eyelid, studies have used borosilicate glass slab [20] and mucin coated glass slab [19] where only the curvature is considered. The exact material properties such as modulus, Poisson's ratio, and density of the ocular structures were not considered. An *in vivo* experiment done on animal models using micro tribometer has led to epithelial damage because of the rubbing of the cantilever against the cornea while estimating CoF [27].

Since *in vitro* studies are not producing reliable results and performing *in vivo* experiments could lead to tissue damage, Finite Element Analysis (FEA) has been used in this study as a tool to analyze the biomechanical changes [16]. FEA is the simulation of any given physical phenomenon using the numerical technique called Finite Element Method (FEM). Using FEA, Kataoka et al. have estimated the deformation and shearing stress between the eyelid and the corneo-conjunctival complex, while considering the ocular surface to be a flat surface [16].

When compared to *in vitro* studies done using micro tribometer, atomic force microscope, etc., FEA is a superior technique for understanding the biomechanical changes (deformation, displacement, and stress) which take place during the blink reflex. However, there are very limited studies [16] that estimate the biomechanical parameters during blinking while considering the actual anatomy and material properties of the eye.

Hence, our study aims to develop a finite element model (FEM) consisting of the lid wiper, eyeball, and contact lens, in order to estimate the biomechanical changes in the cornea and the lid wiper, during a blink, in the presence as well as absence of CL.

## 2. Methods

FEA is a popular method to analyze complex systems. The benefit of using an FEA on complex problems is that geometry can be precisely defined [28]. It is very sensitive such that it can measure even a subtle change in geometry, which is otherwise difficult to pick up with the current instruments such as Optical Coherence Tomography (OCT) and Anterior Segment OCT [29]. Since FEA uses the static equilibrium and theories of elasticity, it is possible to assess a physical system subjected to multiple external forces with regard to stresses, deformation, and strain [30]. Hence, FEA is chosen as the analytical tool.

A computer with 2.80 GHz CPU and 8 GB RAM is used for this experimental study. COMSOL Multiphysics, an FEA tool, is used for the construction and analysis of the human eye model. The COMSOL Multiphysics is used for the FEA because of its ability to provide accurate FE simulation results. The flow of procedure for the FEA includes the following:

- Modeling the geometry.
- Assigning the material properties.
- Meshing.
- Setting boundary conditions.
- Parametric sweep analysis.
- Postprocessing the results.

Ocular parameters of various structures and their material properties were obtained from previous works of literature done on Indian eyes. Linear static analysis was carried out ignoring gravity.

### 2.1. Modeling

It is unrealistic to consider a single eye geometry since the dimensions of the human eye vary from person to person. Here, we have used the average value of each of the ocular parameters of the Indian eyes obtained through a thorough literature search.

**Figure 1:**
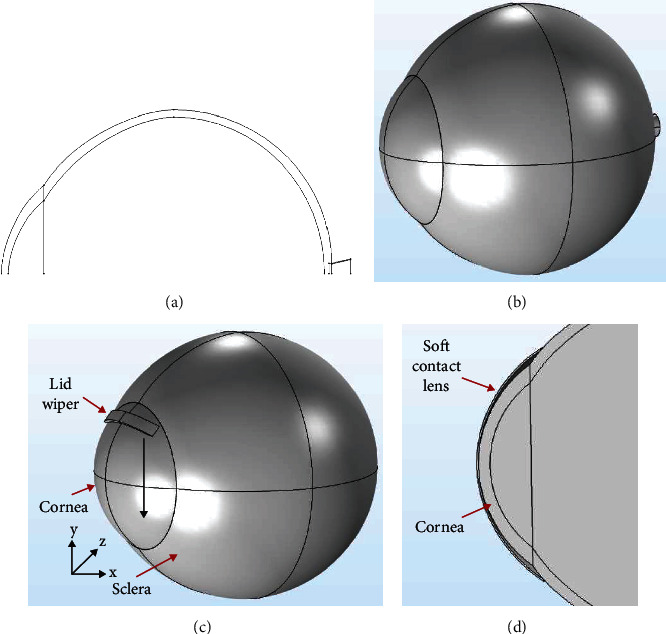
Two-dimensional (a) and three-dimensional model of the eyeball (b). Three-dimensional model of the eyeball with lid wiper (black arrow represents the direction of movement of the lid wiper on the cornea) (c). Cross section of the eyeball with soft contact lens (d). The model was constructed using COMSOL Multiphysics v5.2 (https://www.comsol.com) and the collage of the exported images was made using Microsoft PowerPoint 2019 (https://www.microsoft.com/en-in/microsoft-365/get-started-with-office-2019).

The Indian eye model, which closely resembles the dimensions of the actual human eye, is constructed in 2D (Figure 1(a)) and is converted into a solid 3D model (Figure 1(b)). For simplicity, the human eye is assumed to be rotationally symmetrical along the optic axis. The eye model comprises the following structures: lid wiper, cornea, anterior chamber, vitreous cavity, sclera, optic nerve, and optic nerve head. The anteroposterior (transverse) diameter of the eye model is approximately 24 mm, and the vertical (sagittal) diameter is approximately 23 mm [31].

The asymmetrical nature of the optic nerve is ignored. The dimensions used in the construction of each structure of the human eye are as shown in Table 1.

The prolate nature of the cornea is not considered. A cornea of uniform thickness is assumed for the construction. For the ease of modeling, the scleral thickness and corneal diameter are considered to be uniform throughout the respective structures.

Since eyelid conforms to the curvature of the ocular surface while blinking, we have assumed the curvature of the inner lid wiper to be the curvature of the anterior cornea. The thickness of the lid wiper (*y*-direction, Figure 1(c)) is considered to be 0.8 mm with reference to the literature [32]. The contact width between the lid wiper and cornea (the width of the lid wiper which was in contact with the cornea in the *x*-direction) is 1 mm [37]. The entire simulated model consisting of the eyeball and the lid wiper can be seen in Figure 1(c).

Different soft CL geometries have been explored in previous research [38–40]. CL parameters such as center thickness, base curve, and diameter are available in the literature [41]. In general, a CL is thicker at the center than in the periphery, and this ranges from 0.05 to 0.9 mm. The radius of curvature of the back surface of the lens, i.e., the base curve, generally ranges from 7 to 9 mm, and also the diameter of the CL ranges from 13.00 to 14.50 mm.

In this study, based on the eye geometry, the contact lens of 15 mm diameter (2-3 mm greater than corneal diameter) and 8.6 mm base curve (0.8–1.0 mm flatter than corneal curvature) is fitted in the eye (Figure 1(d)). The peripheral curve radius of soft contact lens (radius of the curve which connects the front and back surface of contact lens) is not available in the literature. Hence, in this study, it is assumed to be 0.5 mm. Contact lens parameters used in this study can be found in Table 1.

Young's modulus, Poisson's ratio, and density are the important material properties considered. Human ocular tissues are generally viscoelastic and exhibit nonlinear material properties [42]. This nonlinear material property of the human eye ranges widely due to its complex nature. These viscoelastic properties of the structures of the eyeball are not yet investigated properly. Hence, the material properties are assumed to be homogenous, isotropic, and linearly elastic. As shown in Table 2, the material properties of the ocular structures are obtained from the previous works of literature. Young's modulus of the human eyelid has not yet been investigated [43]. Hence, a value of 0.42 MPa, which is Young's modulus of the human skin, is assumed [43, 44]. Poisson's ratio of the aqueous humor, vitreous humor, retina, zonules, and optic nerve has not yet been investigated. Since soft biological tissues hold more amount of moisture, Poisson's ratio is set to be less than 0.5 for these ocular structures [42, 45].

The material properties used for the CL are as shown in Table 2. CL is a rubbery polymer, and it is highly hydrated. Hence, a Poisson's ratio of 0.49 was set, which makes it incompressible. Density of the contact lenses is not directly available in the literature. However, specific gravity is available for the contact lenses. Hence, using the following formula, we have calculated the density of these contact lenses.

(1) $$\begin{array}{c} \text{Specific gravity }=\frac{\text{Density of the contact lens }}{\text{Density of the water at }4^{{}^{\circ}}\text{C}}, \end{array}$$

where density of the water at 4°C = 999.97 [55] and specific gravity of the contact lens = 1.04 [56].

### 2.2. Mesh Convergence Study

**Figure 2:**
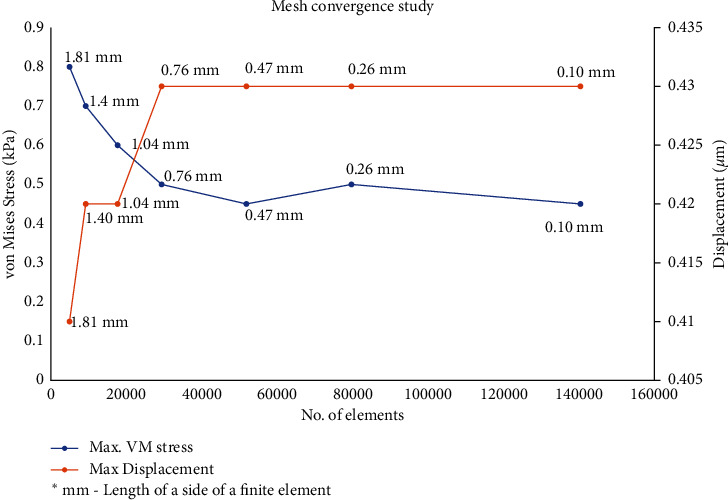
Mesh convergence study: proper number of finite elements. Graph drawn with Microsoft Excel 2019 (https://www.microsoft.com/en-in/microsoft-365/get-started-with-office-2019).

Mesh convergence study was carried out to analyze the proper number of finite elements.  Figure 2 shows the result when a body load of 0.03 N is applied on the surface of the lid wiper. It can be found that the VMS barely changes when the length of a side of the finite element is 0.259 mm or more. Hence, the value of 0.259 mm is used as the length of a side of the element during Finite Element Analysis.

**Figure 3:**
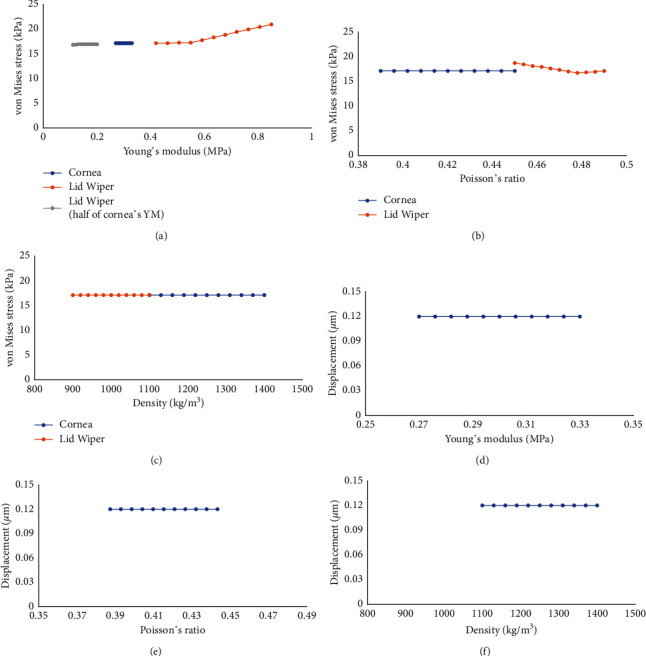
Sensitivity analysis of the material properties of the cornea and lid wiper. Change in Young's modulus of cornea does not have any effect on the VMS of the cornea whereas VMS increases slightly with the increase in Young's modulus of the lid wiper (a). Change in Poisson's ratio of cornea does not have any effect on the VMS of the cornea whereas VMS decreases slightly with the increase in Poisson's ratio of the lid wiper (b). Change in density does not have any effect on VMS of the cornea and lid wiper (c). Change in Young's modulus, Poisson's ratio, and density does not have any effect on displacement of the cornea (d–f). Graph drawn with Microsoft Excel 2019 (https://www.microsoft.com/en-in/microsoft-365/get-started-with-office-2019).

Sensitivity analysis has been carried out to study the effect of material properties (Young's modulus, Poisson's ratio, and density) of the cornea and lid wiper on the outcome parameters (VMS and displacement). Each material property was studied individually by keeping the other two material properties constant. For example, when Young's modulus of the lid wiper was varied between 0.45 and 0.85 MPa, by keeping Poisson's ratio (0.49) and density (999 kg/m^3^) of lid wiper constant, the corresponding change in VMS in the lid wiper was found to be between 17.1 and 20.9 kPa. Our sensitivity analysis results (Figure 3) showed that there was no significant variation in the outcome parameters when the material properties of the cornea and lid wiper were changed. However, there was a slight variation noted in the VMS when Young's modulus of the lid wiper was changed. This is due to the huge variation in Young's modulus of the lid wiper (0.45 MPa to 0.85 MPa). Since Young's modulus of the lid wiper is not available in the literature, the current study has assumed Young's modulus of skin to be Young's modulus of lid wiper. Young's modulus of the skin varies widely across different studies, which explains why there is a slight variation in the VMS in the lid wiper. Since conjunctiva is more flexible than cornea, Kataoka et al. have assumed Young's modulus of eyelid to be half that of the cornea. In a similar assumption, we have also considered Young's modulus of lid wiper to be between 0.10 and 0.20 MPa (half of the cornea), and we have found the corresponding VMS to be between 16.8 and 16.9 kPa. Overall, our sensitivity analysis has shown that the VMS and displacement were not varying significantly based on a single value of the material properties. The detailed values of sensitivity analysis of the material properties of the cornea and lid wiper in terms of von Mises stress and displacement are included in Supplementary Tables 1Sa and 1Sb.

**Figure 4:**
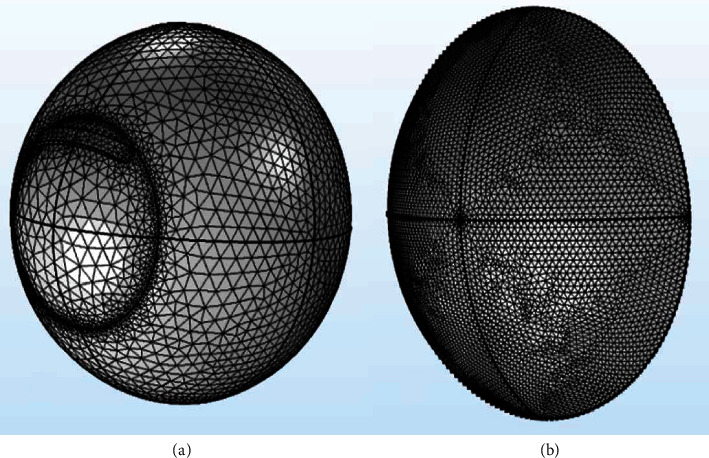
Meshing of the eye (a) and contact lens (b). The model was constructed using COMSOL Multiphysics v5.2 (https://www.comsol.com), and the collage of the exported images was made using Microsoft PowerPoint 2019 (https://www.microsoft.com/en-in/microsoft-365/get-started-with-office-2019).

All components of the FEM are meshed with the physics-controlled settings in the COMSOL Multiphysics. The predefined size of each element is set as “fine.” Figure 4 shows the mesh-divided model of the eyeball with lid wiper (Figure 4(a)) and the contact lens (Figure 4(b)).

### 2.3. Analysis

The outer surface of the sclera is fixed completely. During blink, the lid wiper is in contact with the cornea. Hence, a contact pair was created between these two surfaces. The dynamic friction coefficient of the contact surface between the cornea and the lid wiper was set to 0.1 with reference to the previous literature [57]. The eyelid itself exerts some amount of force over the cornea during blink [10]. Hence, a body load of 0.03 N was applied at the surface of the lid wiper [43]. In order to account for the effect of intraocular pressure (IOP), a boundary load of 15 mmHg (normal IOP of human eye ranges between 10 and 21 mmHg) was applied at the inner boundary of the aqueous humor.

Blink is simulated by making the lid wiper move over the cornea (Figure 1(c)). Parametric sweep analysis is carried out by displacing the lid wiper for every 10°, i.e., from the superior to the inferior portion of the cornea. Linear static analysis dealing with the contact problem was carried out. The whole analysis along with the parametric sweep required approximately 12 hours to complete. VMS and displacement were obtained as a result of FEA. VMS is a scalar value which determines whether a given material will yield (deform plastically) or fracture when a load is applied. In this study, the stress experienced by the cornea as a result of the force exerted by the lid wiper was estimated. Hence, VMS was chosen. In humans, stress represents the feeling of pain. Displacement is the distance at which one object has moved from its original location when an external force is applied. In this study, the displacement indicated how much cornea has moved from its original position as a result of force produced by the lid wiper during blink. This indicates the amount of biomechanical response in human tissues [58]. Surface plots were used to display the results of the analysis.

## 3. Results

**Figure 5:**
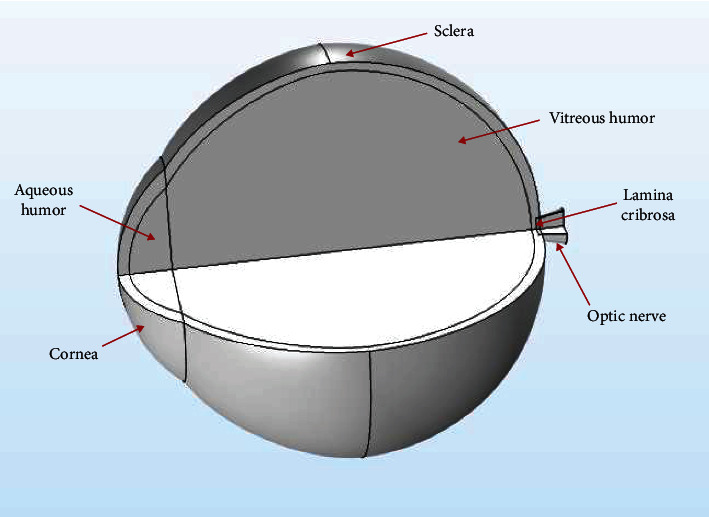
Three-dimensional model of the eyeball depicting the different ocular structures. The model was constructed using COMSOL Multiphysics v5.2 (https://www.comsol.com).

The three-dimensional model of the eyeball (Figure 5) was created using the COMSOL Multiphysics (v5.2, COMSOL AB, Stockholm, Sweden) tool.

The biomechanical changes due to the blink were simulated using the FEM, and the results were obtained as von Mises stress (VMS) and displacement. Initially, the blink was simulated by displacing the lid wiper for every 10° (1.3 mm) using the parametric sweep. All the von Mises stress and displacement values are provided in Supplementary Table 2S.

### 3.1. Without Contact Lens

**Figure 6:**
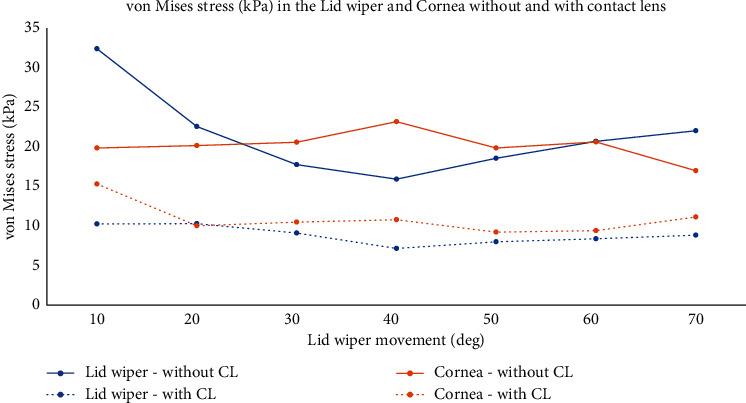
Von Mises stress (kPa) in the lid wiper and cornea during blink without and with CL when the lid wiper is moved at different positions from superior (10°) to inferior cornea (70°) (value of the data labels shows von Mises stress; unit: kPa). Graph drawn with Microsoft Excel 2019 (https://www.microsoft.com/en-in/microsoft-365/get-started-with-office-2019).

**Figure 7:**
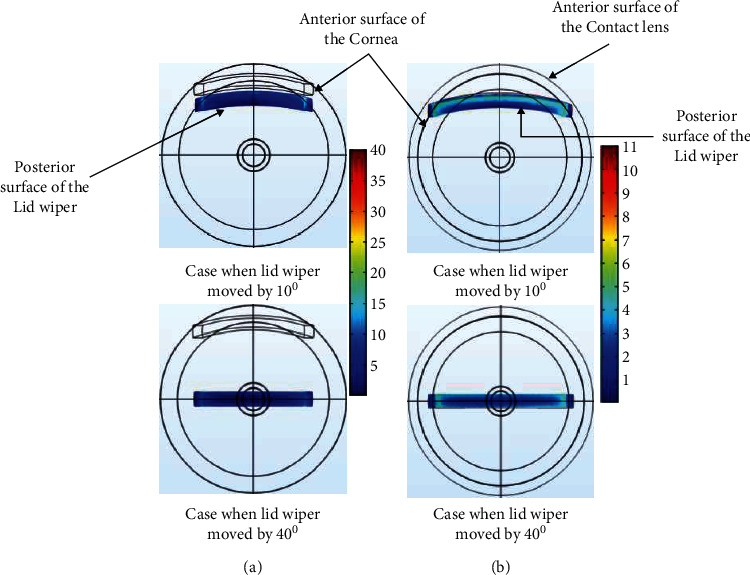
Contour plot of the von Mises stress in the lid wiper during blink without CL (a) and with CL (b) when the lid wiper is moved at superior (10°) and central position (40°) of the cornea (value of the legend shows von Mises stress; unit: kPa). The plots were exported as images from COMSOL Multiphysics v5.2 (https://www.comsol.com), and image collage was made using Microsoft PowerPoint 2019 (https://www.microsoft.com/en-in/microsoft-365/get-started-with-office-2019).

**Figure 8:**
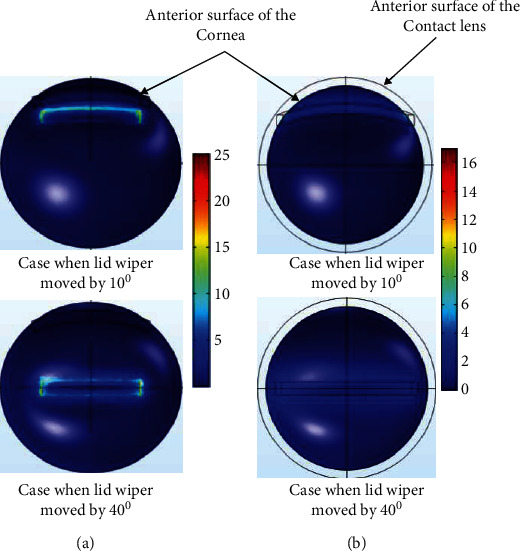
Contour plot of the von Mises stress in the cornea during blink without CL (a) and with CL (b) when the lid wiper is moved at superior (10°) and central position (40°) of the cornea (value of the legend shows von Mises stress; unit: kPa). The plots were exported as images from COMSOL Multiphysics v5.2 (https://www.comsol.com), and image collage was made using Microsoft PowerPoint 2019 (https://www.microsoft.com/en-in/microsoft-365/get-started-with-office-2019).

Figure 6 shows the VMS (kPa) of the lid wiper and cornea at different positions of lid wiper during blink without CL. Lid wiper underwent maximum VMS (32 kPa) when it interacted with the superior 1.3 mm of the cornea (Figure 7(a)). The maximum VMS was seen in the central 1.3 mm of the cornea (23 kPa) compared to the rest of the peripheral cornea during blink (Figure 8(a)). The maximum VMS was noted in the lid wiper (32 kPa) when compared to the cornea (23 kPa) during the interaction between them (Figure 6).

**Figure 9:**
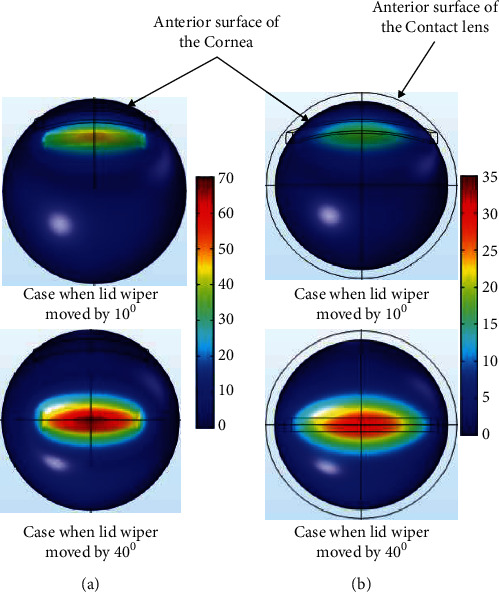
Contour plot of the displacement in the cornea during blink without CL (a) and with CL (b) when the lid wiper is moved at superior (10°) and central position (40°) of the cornea (value of the legend shows displacement; unit: *µ*m). The plots were exported as images from COMSOL Multiphysics v5.2 (https://www.comsol.com), and image collage was made using Microsoft PowerPoint 2019 (https://www.microsoft.com/en-in/microsoft-365/get-started-with-office-2019).

**Figure 10:**
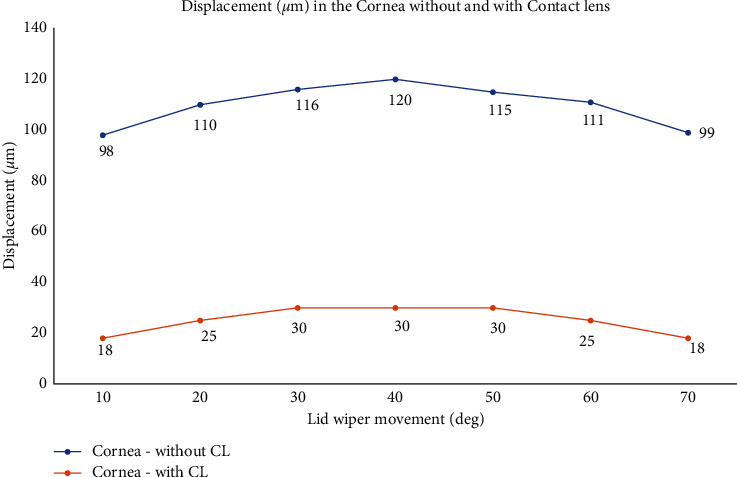
Displacement (*µ*m) in the cornea during blink without and with CL when the lid wiper is moved at different positions from superior (10°) to inferior cornea (70°) (value of the data labels shows displacement; unit: *µ*m). Graph drawn with Microsoft Excel 2019 (https://www.microsoft.com/en-in/microsoft-365/get-started-with-office-2019).

The displacement of the lid wiper was maximum when the lid wiper interacted with the central cornea. Displacement caused by the lid wiper on the cornea during blink is shown in Figure 9(a). It can be seen clearly that the central 1.3 mm of the cornea (120 *µ*m) has been displaced more than the peripheral 2.6 mm of the cornea during blink (Figure 10).

### 3.2. With Contact Lens

Figure 6 shows the VMS (kPa) of the lid wiper and cornea at different position of lid wiper during blink with CL. Lid wiper underwent maximum VMS (10 kPa) when it interacted with the superior 1.3 mm of the cornea (Figure 7(b)). The maximum VMS was seen in the superior 1.3 mm of the cornea (15 kPa) compared to the central 1.3 mm of the cornea (11 kPa) during blink (Figure 8(b)). Maximum VMS was noted in the cornea (15 kPa) compared to the lid wiper (10 kPa) during the interaction between them when CL was *in situ* (Figure 6).

The displacement of the lid wiper was maximum when the lid wiper interacted with the central 1.3 mm of the cornea. Displacement caused by the lid wiper on the cornea during blink is shown in Figure 9(b). It can be seen clearly that the central cornea (30 *µ*m) has been displaced more than the peripheral cornea during blink (Figure 10).

## 4. Discussion

A three-dimensional model of the eyeball was created. The construction of the three-dimensional model of the eyeball used retrospective data from FEA studies [42, 59–62].

### 4.1. von Mises Stress and Displacement: Without Contact Lens

The current study aimed at quantifying the biomechanical changes such as VMS and displacement in the eye during blink. In this study, the VMS was found to be higher in the lid wiper than in the cornea. This large VMS on the lid wiper could be the reason for LWE seen in many patients with ocular dryness and discomfort [10, 14, 16, 63]. This also suggests that the lid wiper would be the first structure to get affected in any ocular surface disorder, even before a change is noticed in the cornea [14, 63]. Hence, the VMS developed during the rubbing could be the reason behind the formation of LWE especially in patients with dry eye disease [10, 64].

VMS was found to be larger in the central 1.3 mm of the cornea than in the peripheral cornea which makes it susceptible to injury by the lid wiper [16]. This is in accordance with the study done by Ousler et al. where they have reported that the ocular surface discomfort is more severe in patients with central corneal damage [65].

Maximum displacement is observed in the central 1.3 mm of the cornea than the peripheral cornea. This suggests that the force produced by the eyelid because of its body load (load that acts throughout the volume of the body) displaces the cornea by 120 *µ*m posteriorly. This finding is in good accordance with the study by Masterton and Ahearne in which they have found out that during blink, the lid wiper pushes the cornea backwards, thereby causing thinning of the tear film, under this compressed area [66]. During blink, the rubbing of lid wiper may cause shear forces on the cornea. This combination of shear forces and displacement caused by the eyelid on the cornea can harm the health of the cornea [66].

One of the most accepted theories proposed by Wright is that the primary factor which causes the development of SLK is constant friction caused by the lid wiper on the conjunctiva and cornea due to excessive laxity of the lid wiper [67]. This has also been proven by one more study where they have found out that in cases of SLK there is upregulation of transforming growth factor-beta 2 (TGF-*β*2) and tenascin 13. These are the factors that are induced by mechanical trauma, thereby proving that mechanical trauma is one of the crucial factors in the development of SLK [52]. Thus, we can attribute the formation of SLK to the large amount of friction caused by the lid wiper on the superior 1.3 mm of the cornea during blink [68].

### 4.2. von Mises Stress and Displacement: With Contact Lens

Studies [10, 66] have proven that the introduction of contact lenses in the eye may disrupt the tear film, thereby dividing the tear film into pre- and post-lens tear film [69]. In some cases, the long-term use of the contact lens can result in the meibomian gland blockage and reduced tear film thickness, thereby causing more friction [70, 71]. This may also lead to the damage of the ocular surface, in some patients [10, 43]. The current study has found that the VMS is larger in the superior 1.3 mm of the cornea than in the central cornea when the CL is in situ. Thus, we can attribute the formation of contact lens-induced SEAL and LSCD to the friction caused by the lid wiper on the superior cornea during blink. In some cases, the inadequate lens flexure creates an area of misalignment in the superior epithelial cornea where pressure from the lid forces the lens against the cornea [72–73]. This produces greater frictional forces on the cornea in the superior region [17, 75]. This is how SEAL occurs in the cornea due to the friction caused by the CL wear [17]. It is reported that about 15.3% of the total LSCD cases are due to CL wear [76]. Mechanical trauma caused by the rubbing of the CL plays a central role in the etiology of CL-induced LSCD [18, 77, 78]. This rubbing is induced by the movement of the soft CL during blink [79]. LSCD due to CL wear is often asymptomatic [80] which makes it is necessary to suspect LSCD in CL wearers.

This study explores the possible mechanism behind the occurrence of certain ocular surface disorders such as SLK, LWE, SEAL, and LSCD in lens wearers and non-lens wearers using FEA. However, there are few limitations in our study. Since the cornea and bulbar conjunctiva follow different curvature, it is difficult to simulate complete blink. Hence, the effect of blink on the bulbar conjunctiva was not considered in this study. Biological systems are quite complex to model and simulate. With the current computing power and modeling and simulation tools available, there are limitations on the exact replication of biological systems, more so when they involve complex tribological aspects. During blink, the pressure exerted by the eyelid on the corneo-conjunctival complex varies depending upon the downward or upward motion of the eyelid. In this study, only the downward motion of the blink process was studied considering challenges with computational resources. Studying the upward phase of the eyelid would give a complete picture of the blinking process. Various theories have been proposed by researchers with regard to the tribological behavior of the human eye with and without contact lens [81]. Researchers say that the behavior is hydrodynamic when a thick tear film is present, and when there is no tear film, the lubrication behavior could be from boundary to mixed regime. Even when there is a tear film present, the tribological behavior could be in the elasto-hydrodynamic regime. In this study, the various regimes of lubrication are not considered, except maybe boundary, wherein there is almost no tear present. In future, studies involving the effect of the tear film and bulbar conjunctiva could give us further insights into the effect of the blink on the ocular surface.

## 5. Conclusion

In this study, we have found that, during blink, the lid wiper has displaced the cornea posteriorly by 30 *µ*m. With the CL in situ, during blink, the lid wiper pushes the CL against the ocular surface, which effectively moves the tears away, causing the surface to dry, thereby creating friction [17]. Hence during blink, the CL slides over the high-pressure areas, i.e., the superior cornea and superior limbus. On long-term use of CL, this can induce significant trauma to the ocular surface [18, 78, 82–85]. We also noted in our study that, during the blink reflex, the lid wiper pressure is less as it approaches the inferior cornea. This is why the CL induces less mechanical trauma at the inferior cornea and limbus [18]. Hence, the occurrence of CL-induced LSCD is also uncommon in the inferior areas. Overall, FEA is a useful simulation tool that helps us to understand the effect of blink on a normal eye with and without CL. This can be extended to other wider areas of research including simulation of surgery and trauma.

## Figures and Tables

**Table 1:** Dimensions of ocular structures and contact lens used in the finite element modeling.

Part of the eye	Value (mm)
Lid wiper thickness [32]	0.8
Corneal thickness [33]	0.5
Corneal diameter [34]	12
Anterior corneal curvature [34]	7.8
Posterior corneal curvature [34]	6.5
Scleral thickness [35]	0.5
Scleral radius [36]	11.5
Diameter of the contact lens chosen	15
Base curve of the contact lens chosen	8.6
Thickness of the contact lens chosen	0.08

**Table 2:** Material properties of the ocular structures and contact lens used in the finite element modeling.

Part of the eye	Young's modulus (MPa)	Poisson's ratio	Density (kg/m^3^)
Lid wiper [44, 46]	0.42	0.49	999
Cornea [30, 47, 48]	0.4	0.42	1400
Aqueous humor [30, 49]	0.037	0.49	999
Vitreous humor [30, 49]	0.042	0.49	999
Retina [45, 50–52]	0.03	0.49	999
Optic nerve [45, 51, 53]	0.03	0.49	999
Comfilcon A contact lens [29, 54]	0.82	0.49	1040

## Data Availability

Previously reported normative data used to support this study are cited at relevant places within the text as references [29–51, 53–56]. The data supporting the conclusion of this study are included within the article and in its supplementary information file.

## Supplementary Materials

Supplementary Materials Table 1S: von Mises stress (kPa) and displacement (*μ*m) in the lid wiper and cornea during blink without and with CL when the lid wiper is moved at different positions from superior (100) to inferior cornea (700). Table 2Sa: sensitivity analysis of the material properties of the cornea and lid wiper (von Mises stress). Table 2Sb: sensitivity analysis of the material properties of the cornea and lid wiper (displacement).
