# Supplementary material for: Finite Element Analysis of Cornea and Lid Wiper during Blink, with and without Contact Lens
Source: J Ophthalmol. 2022 May 17;2022:7930334. doi: 10.1155/2022/7930334 (PMC9129998; doi:10.1155/2022/7930334)
Supplement: Supplementary Materials — Table 1S: von Mises stress (kPa) and displacement (μm) in the lid wiper and cornea during blink without and with CL when the lid wiper is moved at different positions from superior (100) to inferior cornea (700). Table 2Sa: sensitivity analysis of the material properties of the cornea and lid wiper (von Mises stress). Table 2Sb: sensitivity analysis of the material properties of the cornea and lid wiper (displacement). [file 7930334.f1.docx]

**Supplementary Materials**

**Supplementary 1**

**Table 1Sa:** Sensitivity analysis of the material properties of the cornea and lid wiper (von Mises stress)

| **von Mises stress** | | | | | | | | | | | | | |
| --- | --- | --- | --- | --- | --- | --- | --- | --- | --- | --- | --- | --- | --- |
| **Young's Modulus (MPa)** | **Cornea (kPa)** | **Young's Modulus (MPa)** | **Lid Wiper (kPa)** | **Young's Modulus (MPa)** | **Lid Wiper (half of cornea's YM)** | **Poisson's ratio** | **Cornea (kPa)** | **Poisson's ratio** | **Lid Wiper (kPa)** | **Density (kg/m^3^)** | **Cornea (kPa)** | **Density (kg/m^3^)** | **Lid Wiper (kPa)** |
| **0.27** | 17.1 | **0.42** | 17.1 | **0.11** | 16.8 | **0.39** | 17.1 | **0.45** | 18.7 | **1100** | 17.1 | **900** | 17.1 |
| **0.276** | 17.1 | **0.463** | 17.1 | **0.12** | 16.8 | **0.396** | 17.1 | **0.454** | 18.4 | **1130** | 17.1 | **920** | 17.1 |
| **0.282** | 17.1 | **0.506** | 17.2 | **0.13** | 16.9 | **0.402** | 17.1 | **0.458** | 18.1 | **1160** | 17.1 | **940** | 17.1 |
| **0.288** | 17.1 | **0.549** | 17.2 | **0.14** | 16.9 | **0.408** | 17.1 | **0.462** | 17.9 | **1190** | 17.1 | **960** | 17.1 |
| **0.294** | 17.1 | **0.592** | 17.7 | **0.15** | 16.9 | **0.414** | 17.1 | **0.466** | 17.6 | **1220** | 17.1 | **980** | 17.1 |
| **0.3** | 17.1 | **0.635** | 18.3 | **0.16** | 16.9 | **0.42** | 17.1 | **0.47** | 17.3 | **1250** | 17.1 | **1000** | 17.1 |
| **0.306** | 17.1 | **0.678** | 18.8 | **0.17** | 16.9 | **0.426** | 17.1 | **0.474** | 17 | **1280** | 17.1 | **1020** | 17.1 |
| **0.312** | 17.1 | **0.721** | 19.4 | **0.18** | 16.9 | **0.432** | 17.1 | **0.478** | 16.7 | **1310** | 17.1 | **1040** | 17.1 |
| **0.318** | 17.1 | **0.764** | 19.9 | **0.19** | 16.9 | **0.438** | 17.1 | **0.482** | 16.8 | **1340** | 17.1 | **1060** | 17.1 |
| **0.324** | 17.1 | **0.807** | 20.4 | **0.2** | 16.9 | **0.444** | 17.1 | **0.486** | 16.9 | **1370** | 17.1 | **1080** | 17.1 |
| **0.33** | 17.1 | **0.85** | 20.9 |  |  | **0.45** | 17.1 | **0.49** | 17.1 | **1400** | 17.1 | **1100** | 17.1 |

**Table 1Sb:** Sensitivity analysis of the material properties of the cornea and lid wiper (Displacement)

| **Displacement** | | | | | | | | | | | |
| --- | --- | --- | --- | --- | --- | --- | --- | --- | --- | --- | --- |
| **Young's Modulus (MPa)** | **Cornea (mm)** | **Young's Modulus (MPa)** | **Lid Wiper (mm)** | **Poisson's ratio** | **Cornea (mm)** | **Poisson's ratio** | **Lid Wiper (mm)** | **Density (kg/m^3^)** | **Cornea (mm)** | **Density (kg/m^3^)** | **Lid Wiper (mm)** |
| **0.27** | 0.12 | **0.42** | 0.11 | **0.39** | 0.12 | **0.45** | 0.12 | **1100** | 0.12 | **900** | 0.12 |
| **0.276** | 0.12 | **0.463** | 0.11 | **0.396** | 0.12 | **0.454** | 0.12 | **1130** | 0.12 | **920** | 0.12 |
| **0.282** | 0.12 | **0.506** | 0.08 | **0.402** | 0.12 | **0.458** | 0.12 | **1160** | 0.12 | **940** | 0.12 |
| **0.288** | 0.12 | **0.549** | 0.08 | **0.408** | 0.12 | **0.462** | 0.12 | **1190** | 0.12 | **960** | 0.12 |
| **0.294** | 0.12 | **0.592** | 0.08 | **0.414** | 0.12 | **0.466** | 0.12 | **1220** | 0.12 | **980** | 0.12 |
| **0.3** | 0.12 | **0.635** | 0.08 | **0.42** | 0.12 | **0.47** | 0.12 | **1250** | 0.12 | **1000** | 0.12 |
| **0.306** | 0.12 | **0.678** | 0.08 | **0.426** | 0.12 | **0.474** | 0.12 | **1280** | 0.12 | **1020** | 0.12 |
| **0.312** | 0.12 | **0.721** | 0.08 | **0.432** | 0.12 | **0.478** | 0.12 | **1310** | 0.12 | **1040** | 0.12 |
| **0.318** | 0.12 | **0.764** | 0.08 | **0.438** | 0.12 | **0.482** | 0.12 | **1340** | 0.12 | **1060** | 0.12 |
| **0.324** | 0.12 | **0.807** | 0.08 | **0.444** | 0.12 | **0.486** | 0.12 | **1370** | 0.12 | **1080** | 0.12 |
| **0.33** | 0.12 | **0.85** | 0.08 | **0.45** | 0.12 | **0.49** | 0.12 | **1400** | 0.12 | **1100** | 0.12 |

**Supplementary 2**

**Table 2S:** von Mises stress (kPa) and Displacement (μm) in the lid wiper and cornea during blink without and with CL when the lid wiper is moved at different positions from superior (10^0^) to inferior cornea (70^0^)

| **Eyelid movement (deg)** | **Von Mises stress (kPa)** | | | | **Displacement (µm)** | | | |
| --- | --- | --- | --- | --- | --- | --- | --- | --- |
|  | **Without Contact Lens** | | **With Contact Lens** | | **Without Contact Lens** | | **With Contact Lens** | |
|  | **Lid wiper** | **Cornea** | **Lid wiper** | **Cornea** | **Lid wiper** | **Cornea** | **Lid wiper** | **Cornea** |
| **10** | 32 | 20 | 10 | 15 | 46 | 98 | 23 | 18 |
| **20** | 23 | 20 | 10 | 10 | 58 | 110 | 28 | 25 |
| **30** | 18 | 21 | 9 | 11 | 64 | 116 | 31 | 30 |
| **40** | 16 | 23 | 7 | 11 | 67 | 120 | 31 | 30 |
| **50** | 19 | 20 | 8 | 9 | 64 | 115 | 31 | 30 |
| **60** | 21 | 21 | 8 | 9 | 58 | 111 | 27 | 25 |
| **70** | 22 | 17 | 9 | 11 | 47 | 99 | 23 | 18 |
